# Differential expression pattern, bioinformatics analysis, and validation of circRNA and mRNA in patients with arteriosclerosis

**DOI:** 10.3389/fcvm.2022.942797

**Published:** 2022-09-13

**Authors:** Yunyun Liu, Kangjie Wang, Guanhua Li, Zhibo Chen

**Affiliations:** ^1^Department of Gynecologic Oncology, Sun Yat-sen Memorial Hospital, Sun Yat-sen University, Guangzhou, China; ^2^Department of Gynecology, The First Affiliated Hospital of Sun Yat-sen University, Guangzhou, China; ^3^Division of Vascular Surgery, The First Affiliated Hospital of Sun Yat-sen University, Guangzhou, China; ^4^Division of Cardiovascular Surgery, Sun Yat-sen Memorial Hospital, Sun Yat-sen University, Guangzhou, China

**Keywords:** arteriosclerosis obliterans (ASO), circRNA, competing endogenous RNA (ceRNA), bioinformatics analysis, vascular smooth muscle cells

## Abstract

**Background:**

Lower limb arteriosclerosis obliterans (ASO) is the formation of atherosclerotic plaques in lower limb arteries, leading to vascular stenosis and occlusion, and is a major factor leading to lower limb amputation. The ASO seriously endangers the physical and mental health of patients. As living standards improve, the disease tends to occur in younger patients, and the incidence keeps increasing year by year. The circular RNAs (circRNAs) have been found to be tissue-specific, and they play an important role in a variety of diseases, but there are few studies on the pathogenic role and expression of circRNAs in ASOs.

**Method:**

Three diseased arteries from patients with ASO and three healthy arteries from healthy donors were collected for second-generation sequencing, and the pathogenic pathways and possible pathogenic circRNAs related to ASO were screened through bioinformatics analysis. PCR and agarose gel electrophoresis were used to validate the sequencing results. The expression of circRNA-0008706 in human arterial smooth muscle cells (HASMCs) was knocked down using siRNA technology to explore its function.

**Result:**

We identified 480 differentially expressed (DE) circRNAs and 2,997 DEmRNAs. Functional analysis revealed that epithelial-to-mesenchymal transition (EMT), lipid transport, regulation of extracellular matrix disassembly, regulation of cardiac muscle cell proliferation, branched-chain amino acid biosynthetic process, and positive regulation of cell growth and migration were enriched. Based on our previous microRNA array results, we constructed an ASO disease-specific competing endogenous (ceRNA) network. After validation, circRNA-0008706 was selected for functional analysis. Knockdown of circRNA-0008706 significantly suppressed the proliferation and migration phenotype of HASMCs and decreased the BCAT1 expression, which may be due to the specific binding of circRNA-0008706 to microRNA-125b-5p.

**Conclusion:**

This study is the first to compare the circRNA and mRNA expression profiles of ASOs and healthy arterial specimens and to construct a disease-specific ceRNA network for ASOs. This study may provide a new therapeutic target for ASO.

## Introduction

Arteriosclerosis obliterans (ASO) is a common disease causing ischemia in the lower limbs of middle-aged and elderly people, and it has an incidence rate of 17.10% (1, 2). Patients with ASO tend to be younger, which places a heavy burden on patients' families and the national health care system. Currently, pharmacological treatment is not effective. Vascular interventional therapy (balloon dilatation angioplasty, stent angioplasty, etc.) has become the main treatment for ASO (3). Interventional therapy has recently achieved immediate results, but the recanalization rate of arterial stenosis within 1 year is still as high as 30%. Also, approximately 12% of patients are forced to amputate their limbs due to severe necrosis (4). Therefore, it is particularly important to explore the pathogenesis of ASO and to seek better treatments.

Vascular smooth muscle cells (VSMCs) are the major cellular components of the middle layer of the lower limb artery, and they are involved in the development and progression of most vascular diseases (5). The phenotypic transformation of VSMCs from a “resting phenotype” to a highly migratory and proliferative “synthetic phenotype” is currently thought to be the main cause of ASO and restenosis after interventional therapy (6). However, the mechanism underlying the phenotypic transformation of VSMCs remains unclear. Studying the internal mechanisms of uncontrolled proliferation and migration and inhibiting excessive proliferation and migration are two important tools to delay the development of ASO and reduce arterial restenosis after surgical intervention.

CircRNAs are a special class of circular non-coding RNAs that have been identified in recent years. Their aberrant expression has been shown to be associated with many diseases, but little is known about the functions of circRNAs in ASOs. As competing endogenous RNAs (ceRNAs), the circRNAs usually share the microRNA response elements (MREs) of microRNAs with their target genes, so sponged microRNAs cannot bind to and alter the expression of target mRNAs. Chen et al. (7) screened differentially expressed (DE) circRNAs with a non-coding RNA array in proliferative and quiescent human aortic smooth muscle cells (HASMCs) and established a ceRNA crosstalk network. They found five hub circRNAs in the regulatory network. In our study, we sequenced the mid-layers of the arteries from patients with ASO and of the arteries of healthy donors to explore the aberrantly expressed circRNAs and mRNAs in ASO lesions and analyzed the possible mechanisms of pathogenic circRNAs by bioinformatics.

## Materials and methods

### Sample collection

We collected samples from the superficial femoral artery of three healthy donors and the diseased superficial femoral artery of three patients with ASO for sequencing and used the distal external iliac arteries from eight additional healthy donors and also from nine patients with ASO for RNA extraction in subsequent PCR validation. The study followed the guidelines of the Declaration of Helsinki. The basic information of all the patients is detailed in Supplementary Table S1.

### Cell culture

Human aortic smooth muscle cells were cultured in high glucose Dulbecco's Modified Eagle Medium (DMEM) (Corning, America) containing 10% of fetal bovine serum (FBS) (HyClone, USA) and 1% of 100x penicillin/streptomycin solution (HyClone, USA). The cells were cultured at 37°C with 5% CO_2_. Proliferative HASMCs were stimulated with 20 ng/ml PDGF-BB (Propotech, USA) after 24 h.

### RNA and DNA extraction

RNA and genomic DNA of the cells were extracted according to the protocols of the RNA extraction kit (Esscience, China) and the EasyPure Genomic DNA Kit (TransGen, China).

### Quantitative real-time PCR

After measuring the concentration of total RNA using a Nanodrop ND-1000 (Thermo Fisher Scientific), cDNA was reverse transcribed using an Evo M-MLV kit (Accurate Biology, China). Quantitative reverse transcription PCR (qRT–PCR) was employed to test the expression of circRNAs. The primers of circRNAs are listed in Supplementary Table S2.

### Agarose gel electrophoresis

The cDNAs and genomic DNAs were amplified. Agarose gel electrophoresis of PCR products was performed with a 2% gel under 120 V for 30 min.

### Dual-luciferase reporter gene assay

StarBase (https://starbase.sysu.edu.cn/) was employed to predict the binding sites of microRNA in circ-0008706. The two binding sites were mutated and cloned into the p-miR-GLO plasmid (Kidan Biosciences, Guangdong China) to generate the reporter vectors. After cotransfection of miR-125b-5p mimics into HASMCs, the relative luciferase activities were detected using a luciferase kit (Promega, America).

### RNA immunoprecipitation assay

A total of 2 x 10^7^ cells were collected and lysed in an RNA immunoprecipitation **(**RIP) lysis buffer following the protocols of the MAGNARIP kit (Millipore, USA), and a 5-μg anti-AGO2 antibody (Abcam, USA) was incubated with A/G magnetic beads for 1 h. The RIP lysates and magnetic beads were incubated and rotated at 4°C overnight. The RNA was washed and extracted for further tests.

### Western blot and cell migration assay

Western blot assays were performed according to our conventional protocols. Electrophoresis was performed using 25 μg of total protein from each sample on a 10% gel under 80 V for 60 min, and transfer membrane electrophoresis was performed under 300 mA for 90 min. Anti-BCAT1 and anti-β-actin antibodies were purchased from Proteintech. A total of 5 × 104 cells were used for the migration assay for each well of the Transwell chamber.

### Library construction and sequencing

Total RNA was isolated from tissues using TRIzol (Invitrogen, USA) according to the manufacturer's protocol. RNA purity was assessed by ND-1000 Nanodrop and the required A260/280 ≥ 1.8, A260/A230 ≥ 2.0. RNA integrity (RIN) was evaluated by an Agilent 2200 TapeStation (Agilent Technologies, USA) requiring RIN ≥ 7.0. Briefly, rRNAs were removed from total RNA using an Epicenter Ribo-Zero rRNA Removal Kit (Illumina, USA). Then, the RNA was treated with RNase R (Epicenter, USA) and fragmented to approximately 200 bp. Subsequently, the purified RNA fragments were subjected to first-strand and second-strand cDNA synthesis followed by adaptor ligation and enrichment with a low cycle according to the instructions of the NEBNext® Ultra™ RNA Library Prep Kit for Illumina (NEB, USA). The purified library products were evaluated using the Agilent 2200 TapeStation and Qubit® 2.0 (Life Technologies, USA) and then sequenced on a HiSeq 3000. The preprocessing of sequencing reads/quality control raw reads included treatment with Trimmomatic tools (V0.36) to remove adapters. Following the read quality control, the reads were scanned with a four-base wide sliding window, cut when the average quality per base dropped below 15, and dropped to <35% of the initiation read length. Then, the read quality was inspected using FastQC software, and the statistical results were output. Differential expression was assessed by DEseq using the read counts as input. The Benjamini–Hochberg multiple test correction method was used.

### Identification and quantification of circRNAs

Two algorithms, CIRI2 and CIRCexplorer2, were used to detect circRNAs. The reads were mapped to the human reference genome GRCh37/hg19 (http://genome.ucsc.edu/) by BWA-MEM or TopHat. The CIRI2 detects paired chiastic clipping (PCC) signals from the mapping information of the reads by local alignment with BWA-MEM and combines them with systematic filtering steps to remove potential false positives. The CIRCexplorer2 uses TopHat and TopHat-Fusion alignment output to detect circRNAs. If a circRNA can be detected by both methods, it will be considered an identified circRNA. The back-spliced junction reads identified in CIRI2 were combined and scaled to reads per million mapped reads (RPM, bwa-mem mapping) to quantify every circRNA. DEcircRNAs were defined as having a Q-value <0.05.

### Functional annotation enrichment analysis of DE mRNAs in the ceRNA network

Differentially expressed genes (DEGs) (|log2 (fold change)|>1 and Q-value <0.05) were uploaded to Metascape (http://metascape.org/gp/index.html#/main/step1) and Webgestalt (http://www.webgestalt.org/option.php) online software to perform Gene Ontology (GO) classification and Kyoto Encyclopedia of Genes and Genomes (KEGG) pathway enrichment analyses.

### Gene set enrichment analysis

Functional annotation enrichment has some flaws, which may be caused by losing mildly changing genes. Using gene rankings to perform gene set enrichment analysis (GSEA) could solve these issues. We performed GSEA using the “clusterProfiler” package in R software version 4.0.5. The gtf files of KEGG, GO, and hallmarks were downloaded from the GSEA website (http://www.gsea-msigdb.org/gsea/index.jsp).

### Construction of ceRNA networks

Based on our previous studies, 11 microRNAs with clear functions in VSMCs and that were proven to be differentially expressed in ASO were selected to build the ceRNA network. The microRNA-mRNA binding information was acquired in StarBase, which summarizes the binding results from different databases, and we selected mRNAs with binding information in at least three databases. The binding sites of microRNAs on circRNAs were predicted by miRanda, RNAhybrid, and TargetScan. The intersection of the results in each of the three databases was used as the final prediction.

### Visualization of data

The data were visualized in the form of a Sanky plot, a volcano plot, a heatmap, and a dot plot using R packages, including ggplot2, enrichplot, RcolorBrewer, pheatmap, ggalluvial, and dplyr. GraphPad Prism 8 was used to plot histograms. The online software Hanabi (https://hanabi.cn/templates?lang=zh-CN) was used to plot pie charts and cumulative bar charts.

## Results

### CircRNA expression profiles between patients with ASO and healthy donors

**Figure 1:**
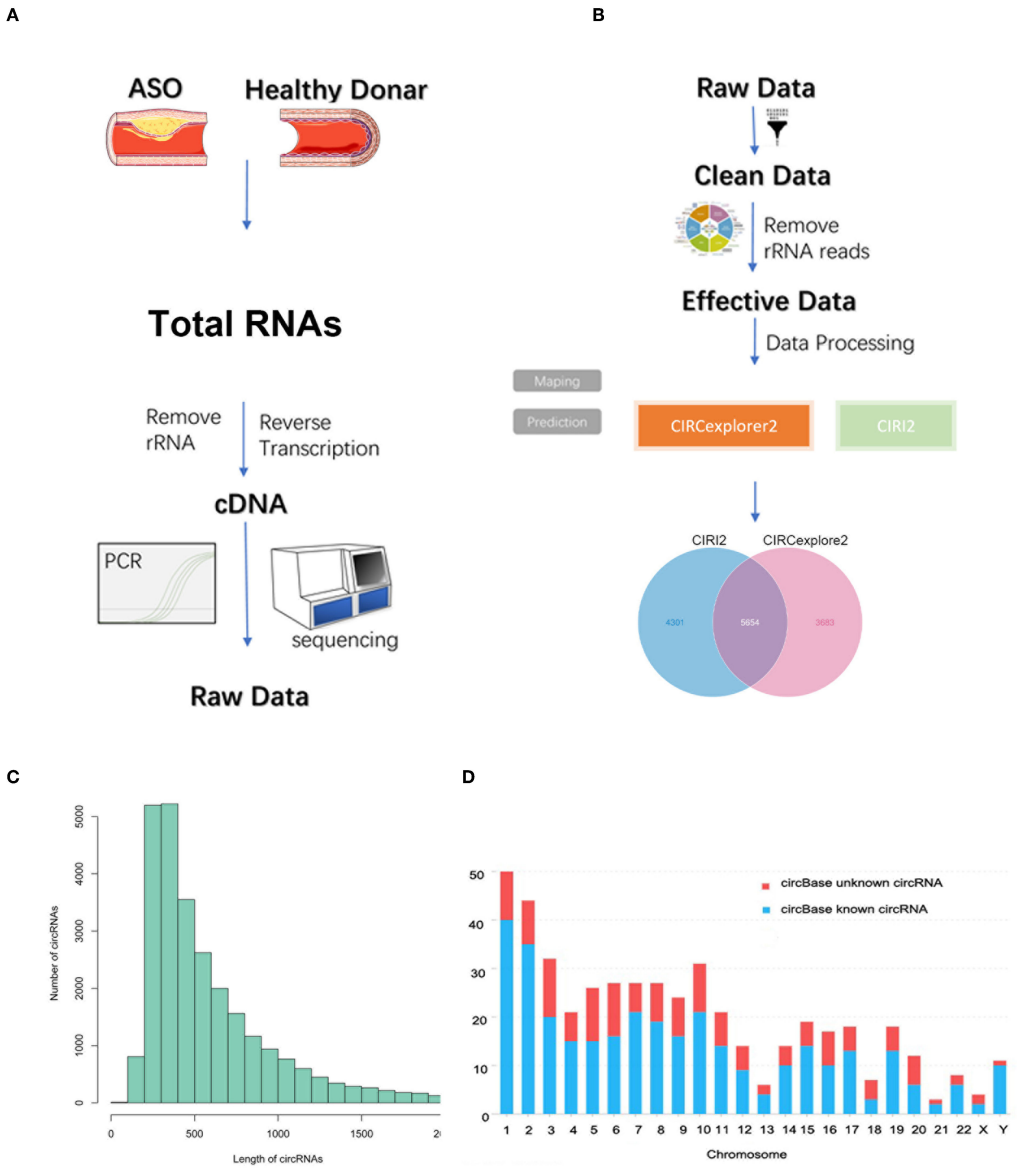
Circular RNA (circRNA) sequencing of the artery tissue workflow. **(A,B)** The workflow of circRNA sequencing using arterial tissues from patients with arteriosclerosis obliterans (ASO) and healthy donors; **(C)** Column distribution map for the lengths of circRNAs; and **(D)** circRNA distribution in each chromosome.

**Figure 2:**
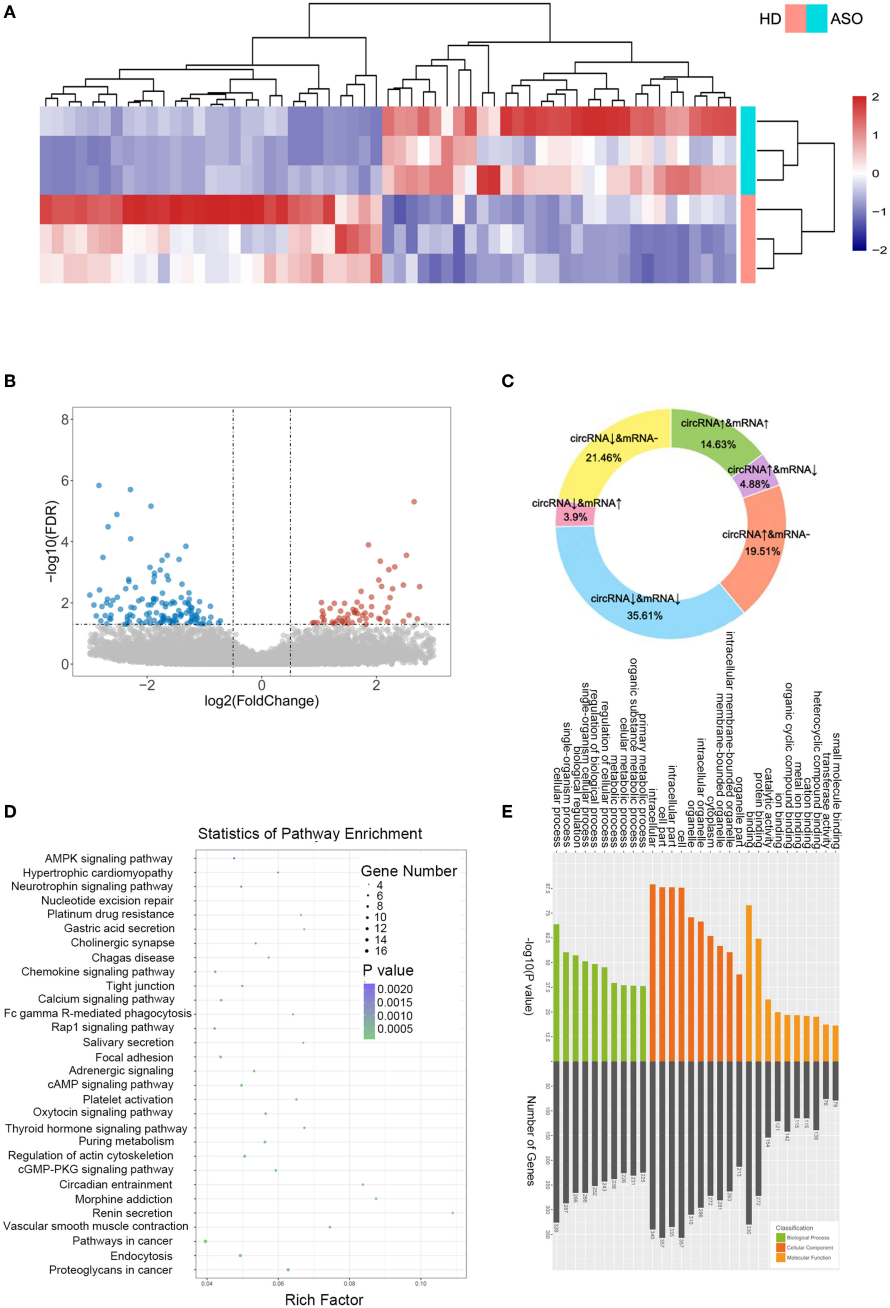
CircRNA expression profiles between patients with ASO and healthy donors. **(A)** A heatmap of the top 60 differentially expressed RNAs (DEcircRNAs) between patients with ASO and healthy donors; **(B)** A volcano plot of all the circRNAs. Blue dots indicate the downregulated circRNAs in patients with ASO, and red dots indicate the upregulated circRNAs; **(C)** A pie chart of the sequencing results; **(D)** A bubble diagram showing the Kyoto Encyclopedia of Genes and Genomes (KEGG) pathway analysis results of DEcircRNAs; and **(E)** GO analysis of DEcircRNAs.

As seen in Figures 1A,B, we used three ASO specimens and three healthy donor tissues for high-throughput sequencing and analyzed the gene expression. CircRNAs were identified using the CIRI2 and CIRCexplorer2 algorithms. The circRNAs identified by both algorithms together were analyzed as candidate genes. Figure 1C shows the length distribution of all identified circRNAs. Figure 1D illustrates the distribution of identified circRNAs in the human chromosome. We identified 480 DE circRNAs, 170 of which were upregulated and 310 of which were downregulated in patients with ASO. The data are shown as a volcano plot and a heatmap (Figures 2A,B). Among these significantly different circRNAs, some failed to match circBase, which might be the newly identified unknown circRNAs.

CircRNAs are special alternative splicing variants of their host genes; the latter can also be spliced into mRNAs. In some circumstances, amplification of a gene may lead to the coregulation of both mRNA and circRNA; similarly, the reduction of a gene could lead to simultaneous downregulation of mRNA and circRNA. Therefore, researchers focused more on the reverse-expressed mRNA and its related circRNA. We summarized the variation trend in circRNAs and their associated mRNAs in Figure 2C. Approximately, half (50.24%) of the DEcircRNAs were coexpressed with their host mRNAs, and 8.78% of the DEcircRNAs were expressed inversely with their host mRNAs. The DEcircRNAs shared no relations with their corresponding mRNAs. Figures 2D,E show the results of KEGG and GO analysis of the DEcircRNAs.

### The mRNA expression patterns in patients with ASO

**Figure 3:**
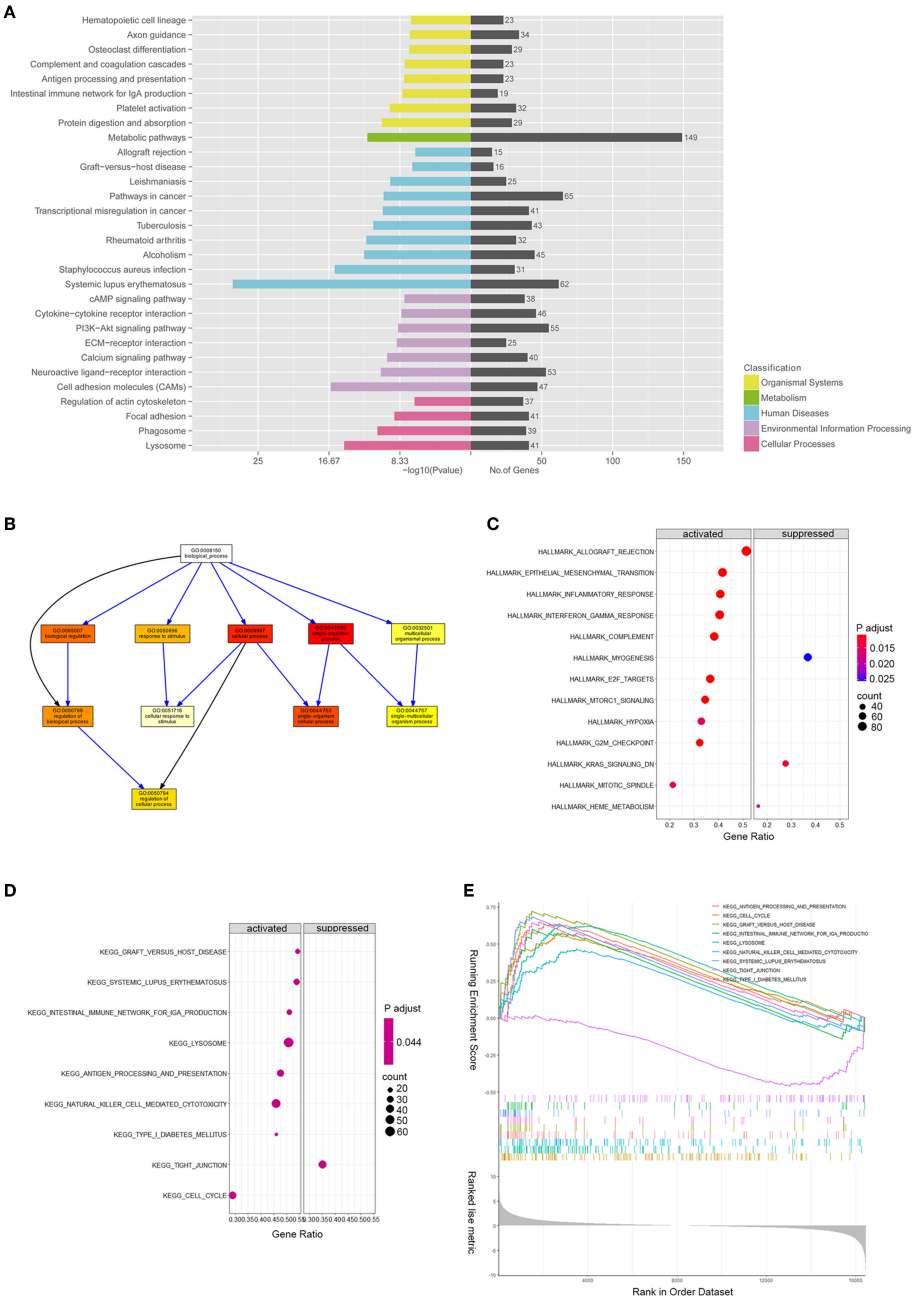
mRNA expression profiles between patients with ASOand healthy donors. **(A)** Kyoto Encyclopedia of Genes and Genomes pathway enrichment results of differentially expressed genes (DEGs) between patients with ASO and healthy donors; **(B)** Directed Acyclic Graph shows the gene ontology (GO) analysis results of DEGs; **(C)** A bubble diagram of gene set enrichment analysis (GSEA) of hallmark pathways; **(D)** A bubble diagram of GSEA of KEGG pathways; and **(E)** GSEA ranking plot of the KEGG enrichment score.

DE mRNAs (|log2 (fold change)|>1 and Q-value <0.05) were used to perform the KEGG and GO analyses (Figures 3A,B). Figure 3A shows the top enriched pathways, including platelet activation, graft vs. host, PI3K-Akt, CAM, and focal adhesion. We found that the vascular smooth muscle contraction pathway, the PPAR pathway, and the HIF-1 signaling pathway were notably enriched. In addition, the enrichment of the glucose metabolic pathways (pentose phosphate pathway, glycolysis pathway, and purine pathway) and the inflammatory pathways (chemokine signaling pathway, MAPK pathway, JAK-STAT signaling pathway, and TGF-beta pathways) were also observed.

### GSEA of DEmRNAs

We ran GSEA enrichment analysis in R, and the gtf files of KEGG, GO, and hallmarks were downloaded from the GSEA website (http://www.gsea-msigdb.org/gsea/index.jsp). In patients with ASO, the hallmark pathways, such as allograft rejection, epithelial-to-mesenchymal transition (EMT), inflammatory response, INFγ, hypoxia, MTORC1, and G2/M checkpoint, were activated. The KEGG pathways, such as graft vs. host disease, diabetes mellitus, nature killer cell-mediated cytotoxicity, and cell cycle were activated (Figures 3C,E), while the KEGG tight junction pathway was suppressed (Figures 3D,E). When epithelial cells undergo EMT, the connections between cells are weakened, which explains why the EMT pathway is activated while the tight junction pathway is suppressed simultaneously. Although VSMCs are not epithelial cells, they may share the common molecular mechanism of epithelial cells when turning into an aggressive phenotype in patients with ASO. The enrichment results from the two databases are mutually supportive, indicating that the enrichment results are more specific.

### Construction of the DEcircRNA-miRNA-DEmRNA ceRNA network

**Figure 4:**
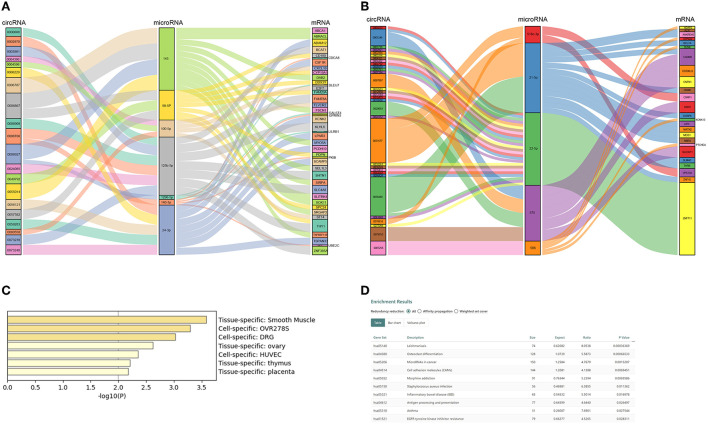
Competing endogenous RNA (ceRNA) network of ASO-related circRNAs and mRNAs. **(A)** A Sanky plot of the ceRNA network in which microRNAs were downregulated in patients with ASO; **(B)** A Sanky plot of the ceRNA network in which microRNAs were upregulated in patients with ASO; **(C)** The tissue distribution result of mRNAs in the ceRNA networks; and **(D)** The pathway enrichment results of mRNAs in the ceRNA networks.

CircRNAs bind to AGO2 proteins to function as ceRNAs. We downloaded the AGO2-binding circRNAs from the circInteractome database (https://circinteractome.irp.nia.nih.gov/). A total of 205 DEcircRNAs binding to the AGO2 protein were used for further analysis (Supplementary Table S3). Our team previously used microRNA arrays to find DE microRNAs between eight patients with ASO and eight healthy donors (8). Further studies confirmed that miR-21-5p, miR-518c, miR-22-5p, miR-575, and miR-586 were upregulated in arteries with ASO, while miR-1298, miR-100-5p, miR-125b-5p, miR-140-3p, miR-98-5p, and miR-24-3p were downregulated in patients with ASO. Therefore, we selected these miRs to build ceRNA networks illustrated in Sankey plots (Figures 4A,B). The circRNAs on the left and the mRNAs on the right are coexpressed (both upregulated or both downregulated) DEGs, and the miRs in the middle are expressed opposite to the neighboring two groups of genes. In addition, we predicted potential or reported combinations of target microRNAs between DEcircRNAs and DE mRNAs. From the interaction network information, we could see that circRNA 0008706 shared MREs of miR-125b and miR-140-3p with 11 mRNAs, including BCAT1, SHNT1, CDCA8, and circRNA-0008927, and could bind miR-145 and miR-1298 competing with 13 mRNAs, including ABCA1, DAB2, FSCN1, and circRNA 0006867, which can bind to miR-145 and miR-98-5p and compete with 17 mRNAs. CircRNA 0031677 shares MREs of miR-21-5p and miR-518c-3p with 15 mRNAs, such as MEIS1, BCL7A, and CSRP1.

### Functional analysis and pathway enrichment of DEmRNAs in the ceRNA networks

There were 67 DEmRNAs in the network. We uploaded these mRNAs to Metascape and Webgestalt for functional analysis. Surprisingly, these genes were enriched in the smooth muscle-specific pathway (PGB00015) (Figure 4C). For the KEGG pathway, the genes were enriched in microRNAs in cancer, cell adhesion molecules, and EGFR tyrosine kinase inhibitor resistance (Figure 4D). The GO-BP analysis showed that positive regulation of EMT and lipid transport, regulation of extracellular matrix disassembly, regulation of cardiac muscle cell proliferation and the branched-chain amino acid biosynthetic process, and positive regulation of cell growth and migration were enriched (Supplementary Table S4). PDPN, DAB2, and FSCN1 positively regulated EMT and extracellular matrix disassembly and were targeted by mir-98-5p and mir-145, respectively. The two microRNAs were both targets of circ-0006867.

### Validation of circRNA

**Figure 5:**
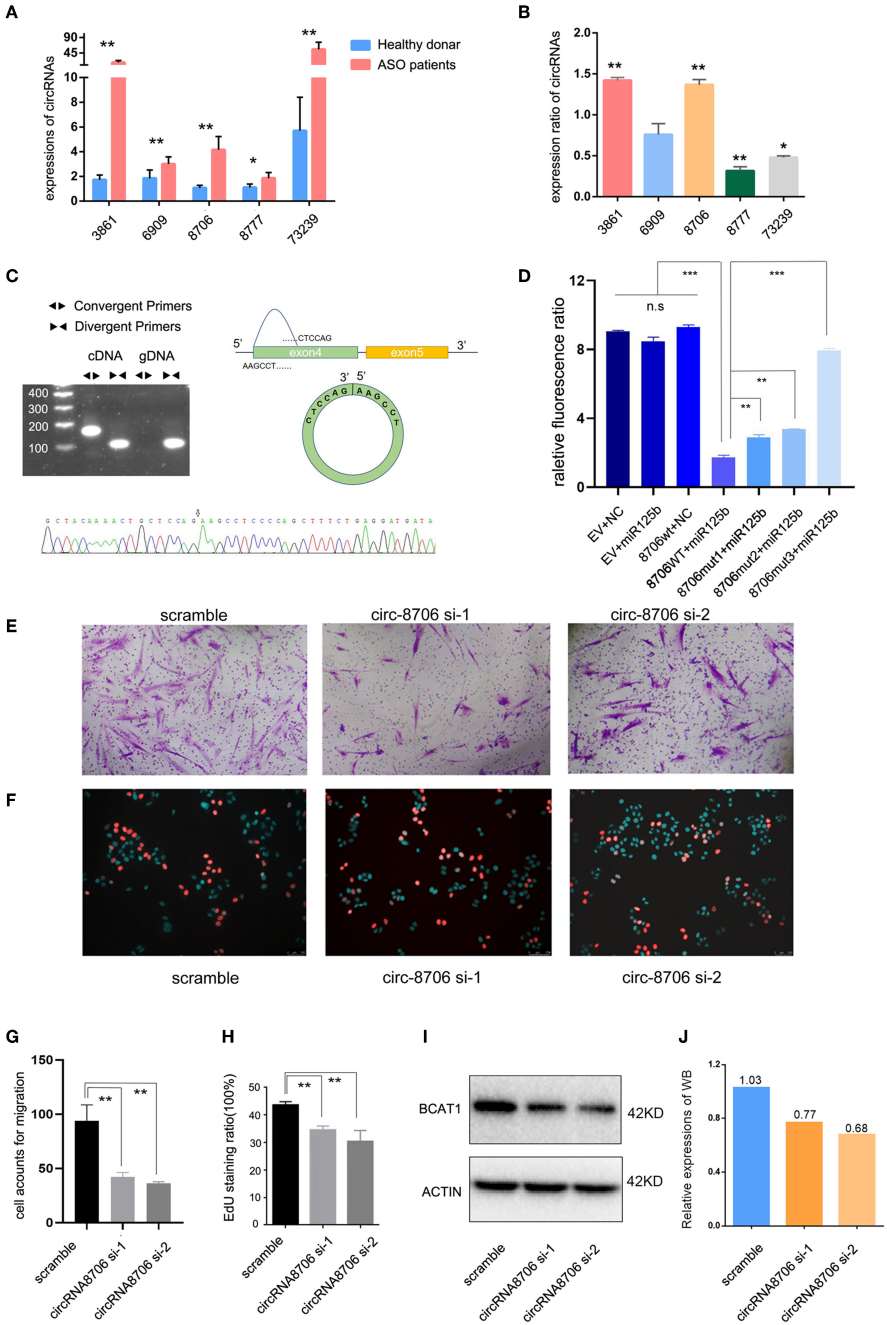
CircRNA-0008706 promoted vascular smooth muscle cell (VSMC) proliferation and migration. **(A)** Validation of circRNA expression in the lesions of patients with ASO (8 ASO specimens and 9 donor specimens); **(B)** Expression ratio of circRNA expression in human arterial smooth muscle cells (HASMCs). The ratio of each circRNA expression after PDGF-bb stimulation/expression without PDGF-bb stimulation; **(C)** Identification of the back-spliced sites of circRNA-0008706; the upper left shows the agarose gel electrophoresis of circRNA-0008706 using cDNA and genomic DNA, the upper right shows the structural representation of circRNA-0008706, and the lower plot shows the Sanger sequence. The black arrow indicates the back-spliced site of circRNA-0008706; **(D)** Relative fluorescence ratio results of the dual-luciferase reporter gene assay. EV, empty vesicles of plasmids; NC, negative control microRNA mimics; wt, wild-type plasmids; mut, mutated plasmids. **(E,G)** Migration assay of HA cells after downregulation of circRNA0008706 (*P* = 0.0011 and 0.007 for siRNA-1 and siRNA-2, respectively); **(F,H)** EdU assay of HA cells after the downregulation of circRNA0008706 (*P* = 0.001 and 0.0053 for siRNA-1 and siRNA-2, respectively); and **(I,J)** Western blot results of BCAT1 before and after the knockdown of circRNA-0008706. *P < 0.05, **P < 0.01, ***P < 0.001.

The author Zhibo Chen has some certain research base in mir-125b; therefore, we chose miR-125b-targeted circRNAs for validation. circRNA-000688, circRNA-0003861, circRNA-0006909, circRNA-0008706, and circRNA-0073239 were selected for qPCR. The RNA extracted from tissues and HASMCs was used to test circRNA expression. We found that circRNA-0008706 and circRNA-0003861 were upregulated in the tissues of both patients with ASO and PDGF-bb-stimulated HASMCs (Figures 5A,B). In contrast, circRNA-73239 and circRNA-8777 were upregulated in patients with ASO but attenuated after PDGF-bb stimulation. Therefore, we selected circRNA-0008706 for the next study because it has two binding sites for miR-125b. RNA pull-down assays showed that circRNA-0008706 could bind to the AGO2 protein (Supplementary Figure S1A). Agarose gel electrophoresis assays and Sanger sequencing were performed to identify the back-spliced sites of circRNA-0008706 (Figure 5C), and RNA stabilization assays confirmed that circRNA-0008706 was stable under Act-D treatment (Supplementary Figure S1B).

### Validation of circ-0008706 binding sites of miR-125b

The StarBase database predicted two binding sites for miR-125b in circ-0008706 (Supplementary Figure S1C). We constructed the wild-type circ-0008706 plasmid and the binding site mutation plasmids (Supplementary Figure S1D). A dual-luciferase reporter gene assay indicated that miR-125b mimics could significantly reduce the luciferase activities of HASMCs transfected with wild-type circ-0008706 instead of mutated circ-0008706 (Figures 5D,J).

### Knockdown of circ-0008706 inhibits the proliferation and migration of HASMCs

We then knocked down the circRNA-0008706 expression with siRNA (Supplementary Figure S1E). EdU assays and Transwell assays were performed. The results revealed that the knockdown of circ-0008706 reduced the proliferation and migration of HASMCs (Figures 5E–H). circRNA-0008706 shared MREs of miR-125b and miR-140-3p with 11 mRNAs in our ceRNA networks, from which BCAT1 plays a vital role in the regulation of branched-chain amino acid metabolism. Furthermore, BCAT1 shared 59 microRNAs with circRNA-0008706. WB assays showed that the knockdown of circRNA-0008706 reduced BCAT1 expression (Figure 5I).

## Discussion

Arteriosclerosis obliterans is a common disease that causes lower limb ischemia and avascular necrosis. Lipid accumulation in the layer of arterial walls, VSMC migration to the intima, and proliferation are the main features of ASOs. As the initial lesions of ASO progress, advanced plaques gradually form and ultimately result in artery stricture or obliteration. In this process, aseptic inflammation increases, local oxygen supply decreases, and VSMC contractility is diminished.

In this study, we performed sequencing using arteries of patients with ASO and healthy donors. Functional annotation of DEmRNAs showed that the vascular smooth muscle contraction pathway, the PPAR pathway, the HIF-1 signaling pathway, the glycolysis pathway, the MAPK pathway, the JAK-STAT signaling pathway, and the TGF-beta pathways were enriched. HIF-1 is a key regulator when cells encounter hypoxic conditions (9). Jain et al. (9) reported that HIF-1α plays a significant role in both the proliferation and migration of VSMCs, leading to cellular dysfunction and inflammation in ASOs. Due to reduced vascular blood flow in ASO lesions, local hypoxia occurs, and hypoxia may induce cells and tissues to convert to glycolysis for energy supply. Studies showed that glycolysis induces VSMC proliferation and leads to ASO (10–12), and the synthetic phenotype of VSMCs indicates increased glycolytic flux and decreased glucose oxidation (13). Peroxisome proliferator-activated receptors (PPARs) are fatty acid sensors that can regulate energy metabolism, including lipid and glucose metabolism (14). PPARs are tissue-specific and are mainly expressed in adipose tissue, macrophages, and muscles. Macrophages absorb lipids and form foam cells in the artery wall in ASOs, so the PPAR pathways are of significance for ASOs. Currently, LY518674 (PPARa agonist) is in the clinical phase II trials to treat ASO (15). Therefore, we believe that the transcriptome sequencing results truly reflect the characteristics of ASO disease and are specific.

**Figure 6:**
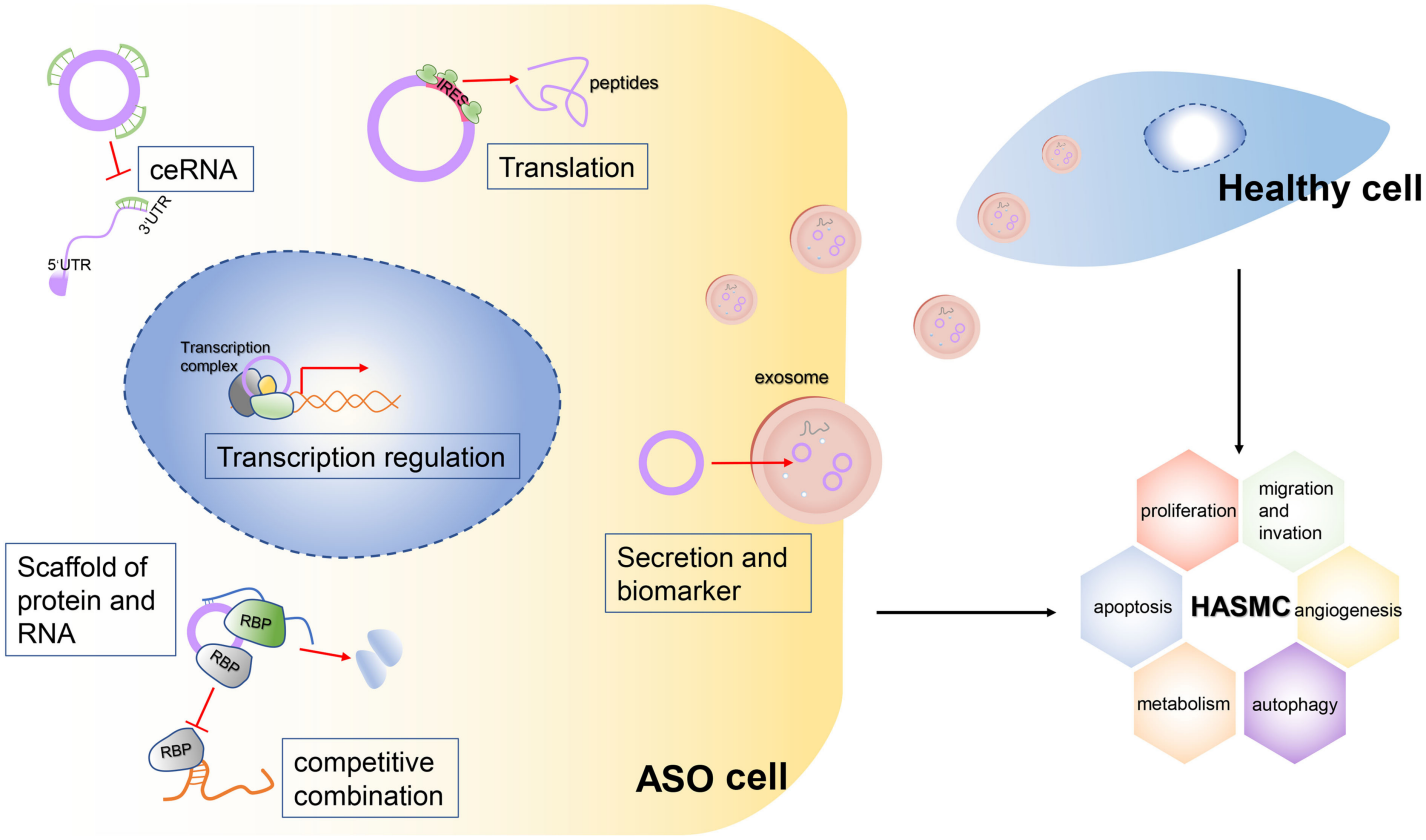
Mechanisms of the potential roles of circRNAs in ASOs.The circRNA plays a vital role in the biological functions of cells. It can act as a sponge for multiple microRNAs, thereby blocking their binding to target RNAs. This mechanism is called the ceRNA mechanism. Second, some circRNAs, especially large circRNAs, competitively bind to certain RNA-binding proteins (RBPs), which inhibit the function of these RBPs. The circRNAs can bind not only to RBPs but also to multiple RNAs, so they can act as scaffolds to facilitate the binding and interaction of some RBPs with their targeted RNAs. Third, some circRNAs have internal ribosome entry sites (IRESs), so they can be translated into peptides and perform biological functions. Finally, circRNAs can be packaged into exosomes and be vehicled extracellularly into the bloodstream, so these circRNAs can transduce signals or can be detected from serum as biomarkers of a certain disease. Meanwhile, secreted circRNAs can be engulfed by healthy cells and stimulate abnormal biological functions of these cells.

CircRNAs are back-splicing products from host precursor mRNAs. They are more stable than linear mRNAs due to the lack of 3' UTRs and their circular structure. Emerging evidence has been raised to suggest the involvement of the ceRNA regulation axis in vascular diseases. Figure 6 shows the mechanisms of circRNAs in ASO. This study is the first to perform circRNA sequencing of ASO arteries and healthy arteries. We aimed to build a ceRNA network in ASOs and identify potential pathogenic circRNAs in ASOs. We found 205 AGO2-binding DEcircRNAs. Then, we selected verified pathogenic microRNAs in ASOs to screen out DEcircRNAs and DEmRNAs. Taken together, we built ceRNA networks in ASOs.

We then performed enrichment analysis using the 67 DEmRNAs in the network. Surprisingly, these genes were enriched in the smooth muscle-specific pathway (PGB00015).

From the interaction network information, we found that circRNA-0008927 and circRNA 0006867 could bind miR-145 and miR-1298 or miR-98-5p, which compete with ABCA1, DAB2, and FSCN1. ABCA1 is annotated as a lipid transport. DAB2 is an adaptor protein that modulates cholesterol homeostasis and uptakes through an LDLR-mediated pathway (16, 17). Furthermore, DAB2 promotes EMT and enhances cell migration and proliferation in ovarian cancer and urothelial carcinoma of the bladder (18, 19), and FSCN1 can positively regulate EMT and extracellular matrix disassembly. CircRNA 0031677 shares MREs of miR-21-5p and miR-518c-3p with 15 mRNAs, such as MEIS1 and CSRP1. The two genes are annotated as muscle tissue development. MEIS1 can promote cell cycle arrest in cardiac muscle tissues and retain cell proliferation.

CircRNA 0008706 shared MREs of miR-125b and miR-140-3p with 11 mRNAs, including BCAT1, SHNT1, and CDCA8. BCAT1 is annotated in the branched-chain amino acid (BCAA) biosynthetic process. During the ASO process, switching of the VSMC phenotype from quiescence to synthesis was recognized. Scientists noticed that amino acid metabolism reprograming can induce phenotypic switches in VSMCs, such as in cysteine, tryptophan, and glutamine metabolism (20–22). However, there are no studies on BCAAs and VSMCs. Sun et al. (23) found that catabolic defects in BCAAs promote heart failure, while enhancement of the BCAA catabolic pathway activity significantly preserves cardiac function. Uddin et al. (24) reported that elevated BCAA levels mediated the mTOR pathway, leading to heart failure. BCAT1 is an enzyme mainly expressed in the cytoplasm that catalyzes the reversible transamination of BCAAs. It can promote cell migration, invasion, and proliferation in many types of cancer (25–27). However, its role in cardiovascular diseases is still unknown.

Theoretically, PDGF-bb stimulation could lead to the phenotypic transformation of VSMCs to simulate the pathophysiologic process of ASO lesions, so we used tissue samples and PDGF-bb-stimulated cells to double validate circRNA expression. Focusing on mir-125b-targeted circRNAs, we found that circRNA-0008706 and circRNA-0003861 were upregulated in the tissues of both patients with ASO and those with PDGF-bb-stimulated HASMCs (Figures 5A,B). CircRNA-73239 and circRNA-8777 were upregulated in patients with ASO but attenuated after PDGF-bb stimulation. Knockdown of circRNA-0008706 inhibited the proliferation and migration of HASMCs. Further luciferase reporter assays confirmed that circRNA-0008706 acted as a sponge for miR-125b-5p.

## Conclusion

For the first time, we established circRNA-microRNA-mRNA networks in patients with ASO using sequencing data. Through enrichment analysis of DEmRNAs in the network, we found that, in addition to glucose and lipid metabolism, amino acid metabolism may also play an important role in ASO, which may have been overlooked previously. Furthermore, the validation results revealed that circRNAs participated in the progression of ASO lesions by directly sponging microRNAs.

## Data availability statement

The datasets presented in this article are not readily available because our university prohibits the uploading of domestic human sequencing data to foreign public databases. Requests to access the datasets should be directed to author ZC, chenzhb23@mail.sysu.edu.cn.

## Ethics statement

The studies involving human participants were reviewed and approved by Ethics Committee of The First Affiliated Hospital of Sun Yat-sen University. The patients/participants provided their written informed consent to participate in this study.

## Author contributions

ZC and GL collected the tissues of patients and wrote the manuscript. YL and KW performed the bioinformatics analyses, designed the primers, and conducted validations and visualization. ZC designed the study. All authors read and approved the final manuscript.

## Funding

This study was supported by the National Natural Science Foundation of China (No.81900428) and the Medical Scientific Research Foundation of Guangdong Province of China (A2022073).

## Conflict of interest

The authors declare that the research was conducted in the absence of any commercial or financial relationships that could be construed as a potential conflict of interest.

## Publisher's note

All claims expressed in this article are solely those of the authors and do not necessarily represent those of their affiliated organizations, or those of the publisher, the editors and the reviewers. Any product that may be evaluated in this article, or claim that may be made by its manufacturer, is not guaranteed or endorsed by the publisher.

## Abbreviations

ASO: arteriosclerosis obliterans
VSMC: vascular smooth muscle cells
ceRNA: competing endogenous RNA
MRE: microRNA response elements
DE: differentially expressed
HASMC: human artery vascular smooth muscle cell
KEGG: Kyoto Encyclopedia of Genes and Genomes
GO: Gene Ontology
GSEA: Gene set enrichment analysis.
