# Supplementary material for: Differential expression pattern, bioinformatics analysis, and validation of circRNA and mRNA in patients with arteriosclerosis
Source: Front Cardiovasc Med. 2022 Sep 13;9:942797. doi: 10.3389/fcvm.2022.942797 (PMC9513155; doi:10.3389/fcvm.2022.942797)
Supplement: Supplementary file 2 [file Data_Sheet_2.docx]

Supplementary Material

# Supplementary Data

Supplementary Material should be uploaded separately on submission. Please include any supplementary data, figures and/or tables. All supplementary files are deposited to FigShare for permanent storage and receive a DOI.

Supplementary material is not typeset so please ensure that all information is clearly presented, the appropriate caption is included in the file and not in the manuscript, and that the style conforms to the rest of the article. To avoid discrepancies between the published article and the supplementary material, please do not add the title, author list, affiliations or correspondence in the supplementary files.

# Supplementary Figures and Tables

For more information on Supplementary Material and for details on the different file types accepted, please see [here](http://home.frontiersin.org/about/author-guidelines#SupplementaryMaterial). Figures, tables, and images will be published under a Creative Commons CC-BY licence and permission must be obtained for use of copyrighted material from other sources (including re-published/adapted/modified/partial figures and images from the internet). It is the responsibility of the authors to acquire the licenses, to follow any citation instructions requested by third-party rights holders, and cover any supplementary charges.

## Supplementary Figures

**Supplementary Figure 1.**

**Legend**:

A: RIP analysis followed by qRT-PCR was applied to assess the AGO2 binding ability of circRNA-0008706 in HASMC. B: RNA stability curve of circRNA-0008706 and its parental gene derived mRNA was performed. 2ug/ml Act-D was used to inhibit nascent RNA. C: the predicted binding sites to miR-125b with circRNA-0008706. D: mutation patterns of circRNA-0008706 overexpression plasmids. E: Relative expressions of miR-125b-5p before and after knocking down of circRNA-0008706. F: Relative expressions of circRNA-0008706 after knocking down through circRNA-0008706 siRNAs.

**Supplementary tables.**

Supplementary Table 1. Clinical data of patients.

Supplementary Table 2. PCR primers and siRNA sequense list.

Supplementary Table 3. List of AGO2 protein binding differential expressed circRNAs.

Legend: the first colomn shows the circRNAs which are predicted to bind to AGO2, the second colomn is the location of each circRNAs, the 3^rd^ colomn named ‘symbol’ is the list of parental genes of circRNAs, colomn ‘control’ shows the mean counts of circRNAs tested using healthy donar arteries, and the colomn ‘treat’ indicates mean counts of circRNAs in ASO speciments.

Supplementary Table 4. Results of GO enrichment analysis of the mRNAs in the ceRNA networks.
