# Supplementary figures and images for: Differential expression pattern, bioinformatics analysis, and validation of circRNA and mRNA in patients with arteriosclerosis
Source: Front Cardiovasc Med. 2022 Sep 13;9:942797. doi: 10.3389/fcvm.2022.942797 (PMC9513155; doi:10.3389/fcvm.2022.942797)

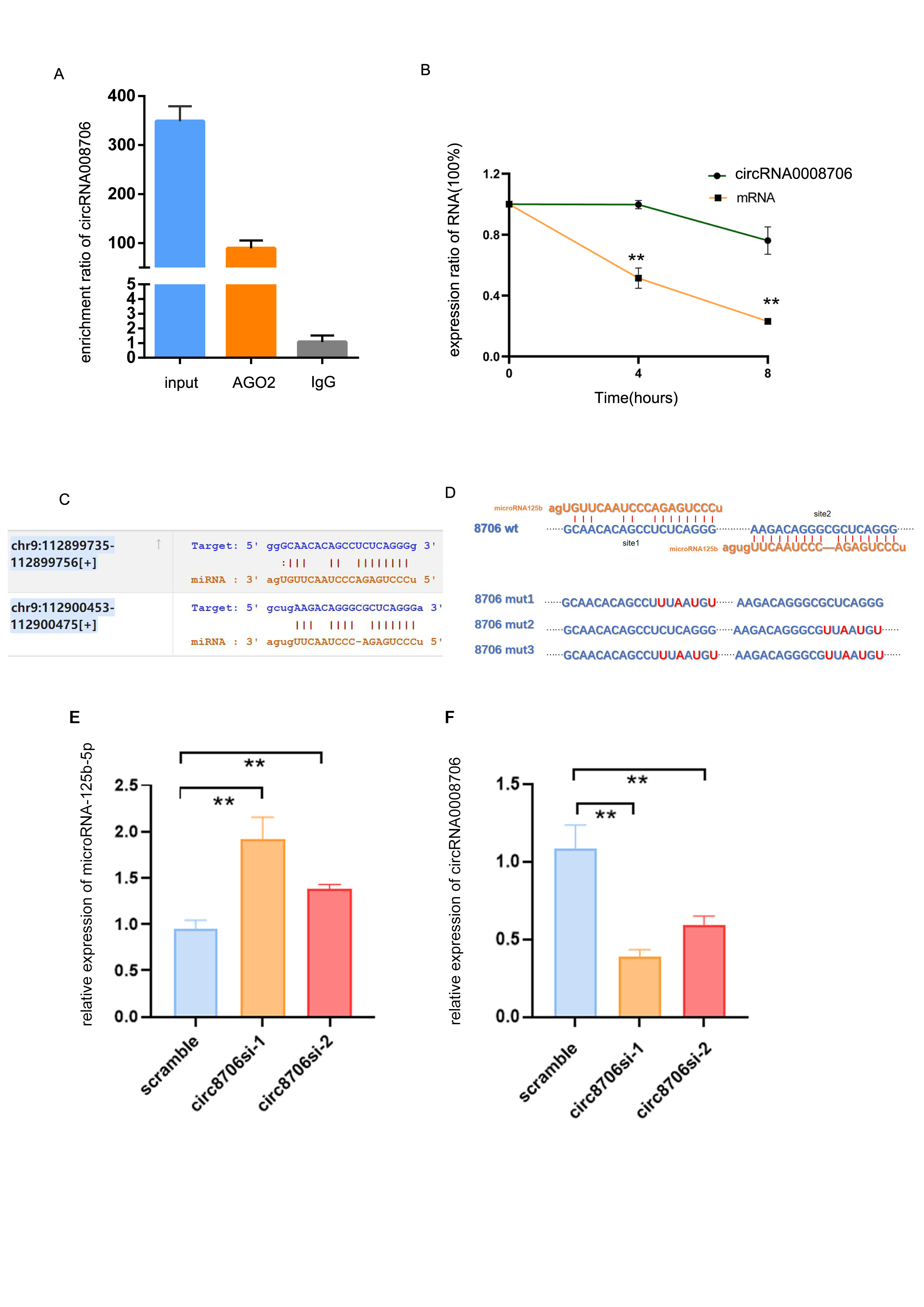

Supplement: Supplementary file 3 [file Image_1.TIF]
